# Patterns of flood preparedness and perception in a marginalised community in the Sol Plaatje Municipality, in the Northern Cape

**DOI:** 10.4102/jamba.v18i1.1973

**Published:** 2026-09-16

**Authors:** Tlou D. Raphela, Refilwe S. Moremane

**Affiliations:** 1 Disaster Management Training and Education Centre for Africa, Faculty of Natural and Agricultural Sciences, University of the Free State, Bloemfontein, South Africa

**Keywords:** floods, risk reduction, preparedness, perceptions, at-risk communities

## Abstract

Flooding poses a growing threat to South Africa’s urban poor, yet limited research has examined how socio-demographic factors and governance intersect to shape household preparedness in marginalised townships. This study addresses that gap by analysing flood preparedness and risk perception in Green Point, a historically under-resourced settlement within the Sol Plaatje Municipality. Data were collected from 114 households through structured questionnaires and open-ended responses, using a mixed-methods design. Quantitative analysis combined chi-square tests, a random forest classifier and multiple correspondence analysis to identify the key socio-economic predictors of preparedness, while qualitative data were thematically analysed to capture the lived experiences and perceptions of municipal performance. Education and employment strongly determined preparedness (*χ*^2^ = 15.25, *p* = 0.004), whereas perceptions of institutional readiness showed weak predictive power. Respondents cited blocked drainage, waste mismanagement and weak municipal coordination as core drivers of recurrent floods. The novelty of this study lies in integrating statistical and perceptual evidence to reveal that vulnerability is socially patterned and governance dependent. Findings underscore the need for equity-focused disaster risk-reduction strategies in under-resourced municipalities, including targeted risk communication, community-based planning and infrastructure investment aligned with local socio-economic realities.

**Contribution:**

Overall, the study finds that flood risk in Green Point is not only a function of environmental exposure but also systemic governance and socio-economic exclusion. Residents are aware of the risks and are willing to contribute to solutions, yet structural support is lacking. The study concludes with targeted, equity-focused recommendations for policy and community engagement in DRR.

## Introduction

Climate change is increasingly being linked to the intensified hydro-meteorological hazards worldwide, including floods that threaten urban communities (Chiappini, Nieri & Piserà 2025; Dodman et al. 2022). Global evidence proves that rising temperatures and shifting rainfall patterns amplify flood frequency and severity, particularly in developing regions with fragile infrastructure (Broekman, Coetzee & Cilliers 2025; Byaruhanga, Kibirige & Mkhonta 2025; Raphela & Matsididi 2025). These climate-driven dynamics necessitate proactive disaster preparedness to protect vulnerable populations. In South Africa, flood impacts are exacerbated by rapid urbanisation, informal settlement growth and under-maintained drainage systems (Mshelia & Belle 2024). Municipalities often face governance and resource constraints that hinder flood-risk reduction. The Sol Plaatje Municipality, encompassing Kimberley and the marginalised settlement of Green Point, has experienced recurrent floods causing property damage, livelihood disruption and heightened community vulnerability.

Existing South African literature has largely examined flood exposure and vulnerability patterns at provincial or regional scales but offers limited insight into the socio-demographic and perceptual determinants of preparedness at the community level (Nyam et al. 2024; Wen & Shaw 2025). Few studies integrate the quantitative and qualitative perspectives to investigate how education, employment and institutional trust interact to shape readiness in low-income urban contexts. By analysing patterns of flood preparedness and perception in the Green Point community, this study addresses that gap. This study contributes to theory by linking the social vulnerability and behavioural dimensions of risk to municipal governance performance and to practice by providing evidence to inform localised, equity-focused interventions for disaster risk-reduction (DRR) within under-resourced municipalities. This study assesses these patterns of flood preparedness and perception in the marginalised Green Point community in the Sol Plaatje Municipality, in the Northern Cape province of South Africa, by addressing the following objectives:

- Assessing the level of household flood-disaster preparedness in the Green Point community within the Sol Plaatje Municipality
- Evaluating the knowledge, perceptions and behaviours of residents regarding storm water drainage systems and their role in flooding
- Examining the effectiveness of municipal flood-risk communication strategies and community awareness mechanisms
- Identifying socio-demographic factors influencing the preparedness for and perception of flood risks among residents

## Research method and design

This study employed a mixed-methods research design, combining the quantitative and qualitative approaches to capture both measurable patterns and lived experiences of flood preparedness in the Green Point community. The approach enabled triangulation between statistical associations and community narratives, thereby increasing the validity and depth of interpretation. A purposive sampling strategy was applied to select 114 respondents representing diverse age groups, genders and household structures within Green Point. This method was deemed appropriate because reliable household listings were unavailable, and community accessibility constraints limited random sampling. Purposive selection ensured the inclusion of households directly affected by previous flooding, thereby enhancing the analytical relevance of the study to disaster risk. While the sample size (*N* = 114) is modest, it exceeds the minimum thresholds for exploratory inferential statistics with categorical data and machine-learning models using multiple predictors (Hair et al. 2019).

To ensure data reliability and instrument validity, the questionnaire was adapted from existing flood preparedness and vulnerability surveys (e.g. Fatemi et al. 2017; Wang, Zhang & Li 2017) and reviewed by two disaster-management specialists for content relevance and clarity. A pilot test with 10 respondents was conducted in a neighbouring settlement, leading to refinement of the question wording and response categories. All participants provided informed consent before data collection. Anonymity and confidentiality were assured, and participation was voluntary. Quantitative data were analysed by using descriptive statistics, Chi-square tests, random forest classification and multiple correspondence analysis (MCA), while qualitative responses were thematically analysed by following Braun and Clarke (2006).

Data for this study were visualised by using a word cloud and a thematic network diagram (Attride-Stirling 2001). All analyses were conducted by using Python 3.11, leveraging packages such as pandas, scikit-learn, matplotlib, seaborn and network.

### Data analysis

Data analysis was designed to respond to the study’s four objectives. To examine whether socio-demographic characteristics are associated with flood-related knowledge and perceptions in the Green Point community, chi-square tests of independence were applied to a series of demographic variables paired with other variables that could predict flood preparedness. The study further identified the key factors influencing whether households in the Green Point, Sol Plaatje Municipality, possess assured means of sustenance, such as savings or insurance, in the event of a flood disaster.

The study employed a random forest classifier for its robustness with categorical predictors and small samples. Flood preparedness was set as the dependent variable, operationalised as whether the respondents had any assured means of sustenance during a flood (Yes = 1; No = 0). Independent variables included age group, education level and employment status, as well as beliefs that drainage systems are a cause of floods and that the municipality is prepared for floods. Categorical variables were label encoded, and the data were split into training and testing sets (70/30). A random forest classifier (*n* = 100 trees) was trained and evaluated on the test set.

To gain deeper insight into the community’s perceptions of flood causes, municipal actions, community roles and social support structures, four open-ended questions were analysed thematically. Responses were grouped by using a coding process based on frequently occurring themes. The most common phrases were categorised under thematic headings. A word cloud was generated to visualise frequent terms, and a thematic network diagram was developed to map the grouped codes to broader themes. To uncover patterns and associations between key categorical variables related to flood preparedness and perceptions among residents of Green Point, the MCA was used to visually and analytically explore the multidimensional relationships among the following:

- Education level × knowledge of drainage systems
- Belief that the municipality is doing enough × perceived flood severity
- Race × access to sustenance during floods
- Age × mode of flood-risk communication

Because of platform constraints, MCA was approximated by using principal component analysis applied to one-hot encoded categorical data. This technique captures the distances between categories in a reduced-dimensional space, revealing clusters and associations. All rows with missing values were removed. Two components were extracted and plotted to observe the relationships.

### Ethical considerations

Ethical clearance was obtained from the University of the Free State Research Ethics Committee (UFS-HSD2022/1299/22), and municipal permission for community access was granted by the Sol Plaatje Disaster Management Unit.

## Results

### Socio-demographic characteristics associated with flood-related knowledge and perceptions

The Chi-squared test showed that both men and women exhibited similar levels of understanding about what constitutes a disaster, indicating that gender is not a significant factor in disaster awareness in this community. Most residents, regardless of race, reported experiencing flooding. A slightly higher proportion of mixed-race respondents indicated flood issues. Education statistically predicted the understanding of drainage systems in the study community (*X*^2^ = 15.25; *df* = 4; *p* = 0.004). Respondents with grade 12 and tertiary education were much more likely to understand stormwater drainage systems than those with primary or no schooling (Table 1). These results indicate that education significantly influences the technical knowledge related to flood risk.

**TABLE 1:** Chi-squared test output for socio-demographic characteristics associated with flood-related knowledge and perceptions in the Green Point community.

Test	Chi^2^ Statistic	*df*	*p*-value
Gender × understanding disaster	0.00	1	1.000
Race × flood experience	3.81	2	0.149
Education × drainage knowledge	**15.25**	**4**	**0.004**
Age × municipal preparedness	4.77	5	0.445

Note: Significance levels are indicated as follows: *p* < 0.05 = statistically significant; *p* < 0.01 = highly significant. Boldface *p*-values denote significant relationships.

*df*, degrees of freedom.

Most respondents across all age groups believed that the municipality was not prepared for floods, as no age group showed significantly more optimism than others (Table 1). Perceived municipal unpreparedness is a universal concern across age groups.

### Predicting flood preparedness using a random forest model

The random forest classifier achieved an overall accuracy of 0.82 on the test set, with an F1 score of 0.76 and ROC-AUC of 0.84. These values indicate good model performance despite the small dataset. Employment status emerged as the most influential predictor, followed by education level and age, confirming that socio-economic stability enhances household flood preparedness. Perceptions of institutional readiness and infrastructure adequacy had minimal predictive weightage, suggesting that personal capacity exerts greater influence than trust in municipal systems. These findings align with international studies linking socio-economic security and adaptive capacity (Fatemi et al. 2017; Wang et al. 2017). Nevertheless, employment status emerged as the strongest predictor, confirming that income stability increases flood preparedness. Additionally, age and education were also key drivers, suggesting that older or more educated individuals are more aware or equipped to prepare for flood disasters. Furthermore, beliefs about institutional preparedness or infrastructure mattered less, indicating that personal capacity plays a larger role than the perception of government readiness (Figure 1).

**FIGURE 1:**
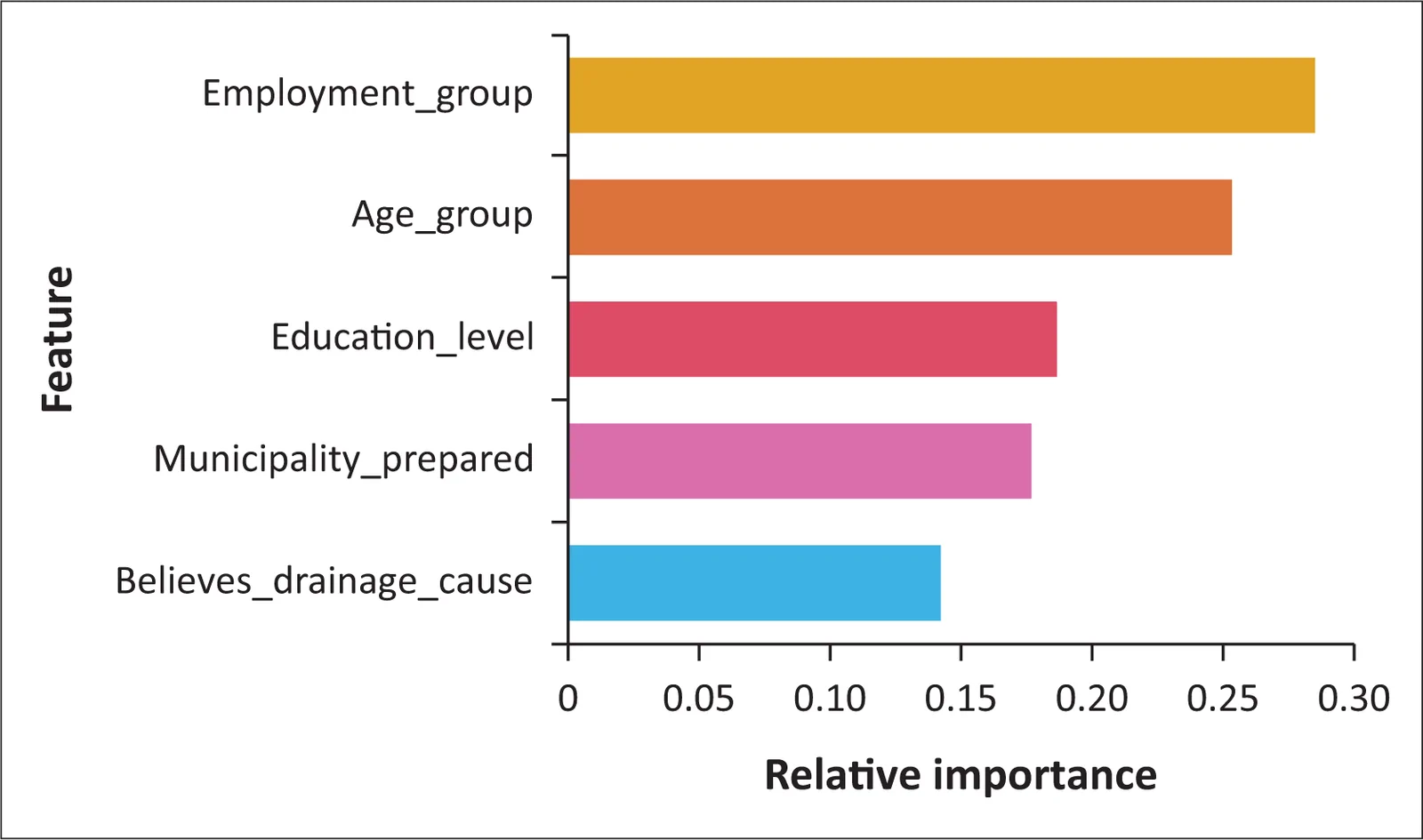
The importance of features in predicting flood preparedness.

### The community’s perceptions of flood causes, municipal actions, community roles and social-support structures

The most frequent words across all responses included ‘drainage’, ‘build’, ‘flooding’, ‘community’, ‘stop dumping’, ‘municipality’, ‘blocked’, ‘infrastructure’, ‘church’ and ‘school’. These reflect widespread concern about infrastructure deficits, uncollected waste and weak institutional or communal coordination as per the word cloud below (Figure 2). Respondents’ narratives underscored infrastructural neglect and waste mismanagement: ‘The drains are always blocked; even small rain floods our street because no one cleans them’ (R23). Others highlighted community co-responsibility: ‘We dump rubbish ourselves; we also need to change and work with the municipality’ (R41). Such reflections demonstrate awareness of both structural and behavioural drivers of flood risk and reinforce the quantitative findings.

**FIGURE 2:**
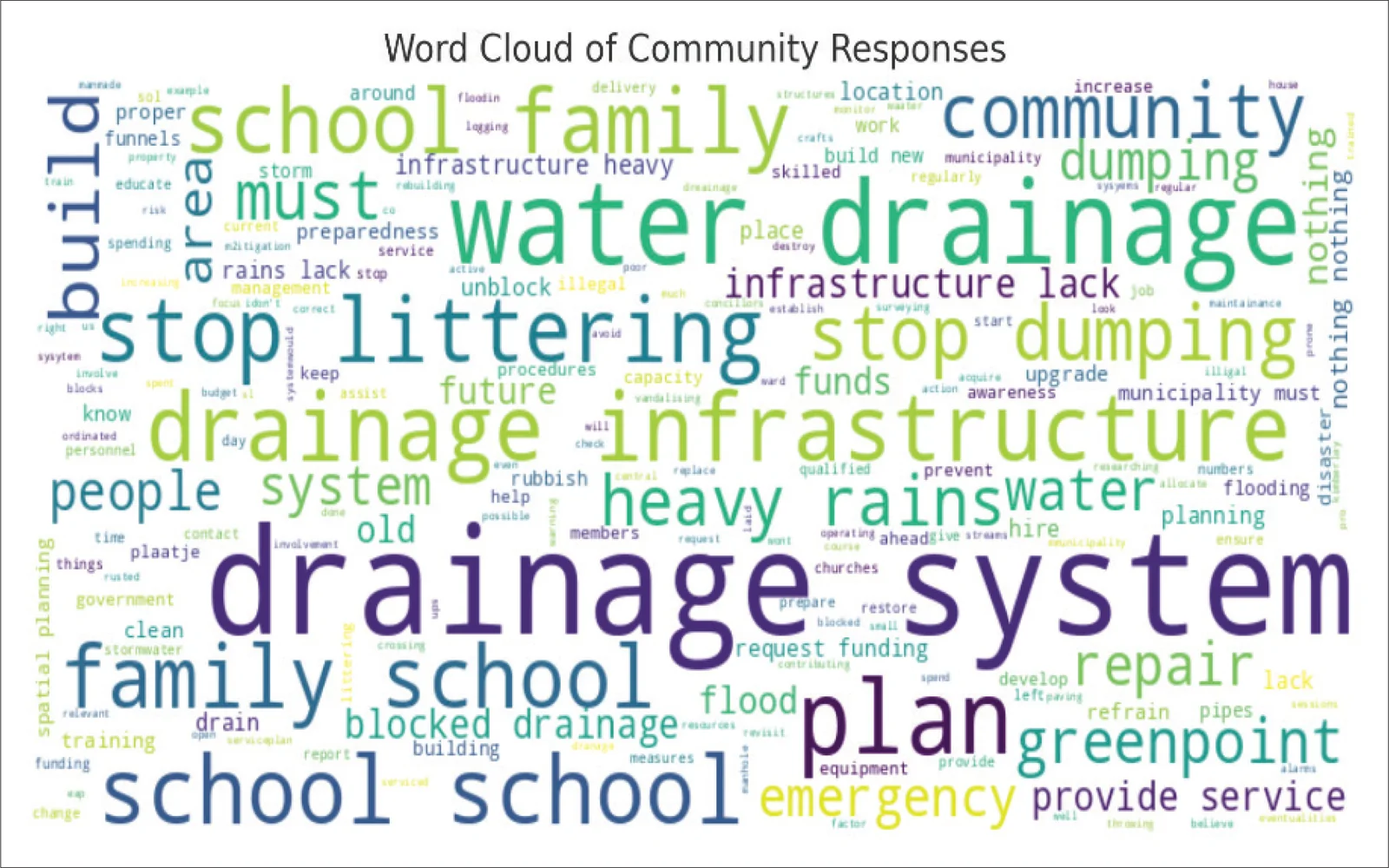
Word cloud of community responses on perceptions of flood causes, municipal actions, community roles and social-support structures.

Each theme was mentioned consistently across responses. Infrastructure failures were perceived as the root cause, while both the municipality and the community were viewed as having a role in mitigating risks, as shown in the thematic network findings below (Figure 3).

**FIGURE 3:**
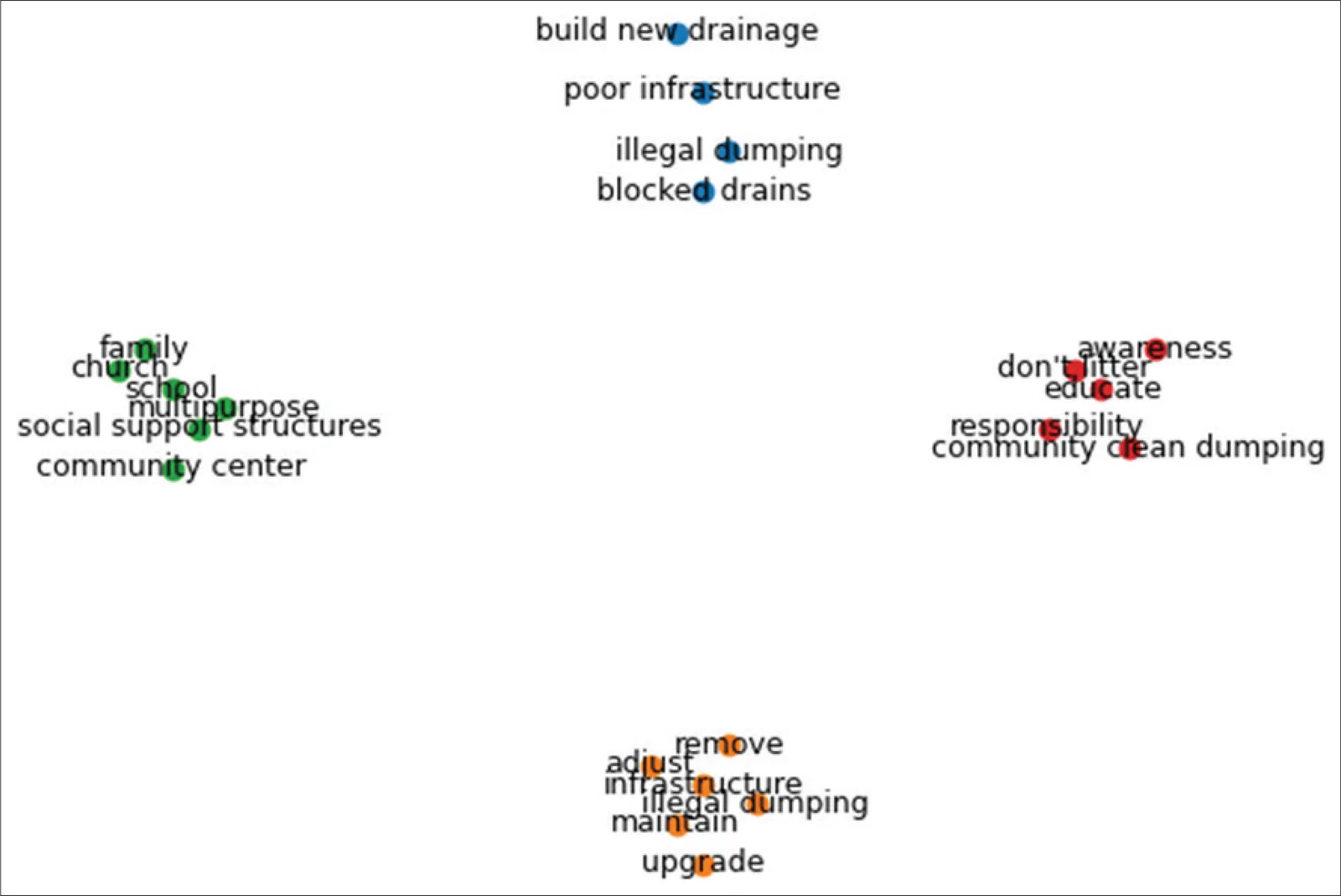
Thematic network diagram for community perceptions and solutions.

### Patterns and associations between key categorical variables related to flood preparedness and perceptions among residents of Green Point

The MCA scatter projects the encoded data into a 2D space. Each point represents a respondent, with their categorical characteristics influencing their location (Figure 4). The two principal components extracted through the MCA proxy accounted for 57.8% of the total variance, indicating that the two-dimensional representation adequately captured key relationships among categorical variables. The education level and drainage-system knowledge formed a strong positive cluster, supporting Chi-square results that link technical understanding with formal education. Race and sustenance patterns also co-clustered, with mixed-race respondents more likely to report modest savings or insurance. Perceptions of municipal preparedness remained widely dispersed across the plot, signalling inconsistent institutional performance and variable community trust. The divergence in communication modes between younger and older respondents – interactive versus passive – illustrates a generational gap in information access critical for future DRR strategies.

**FIGURE 4:**
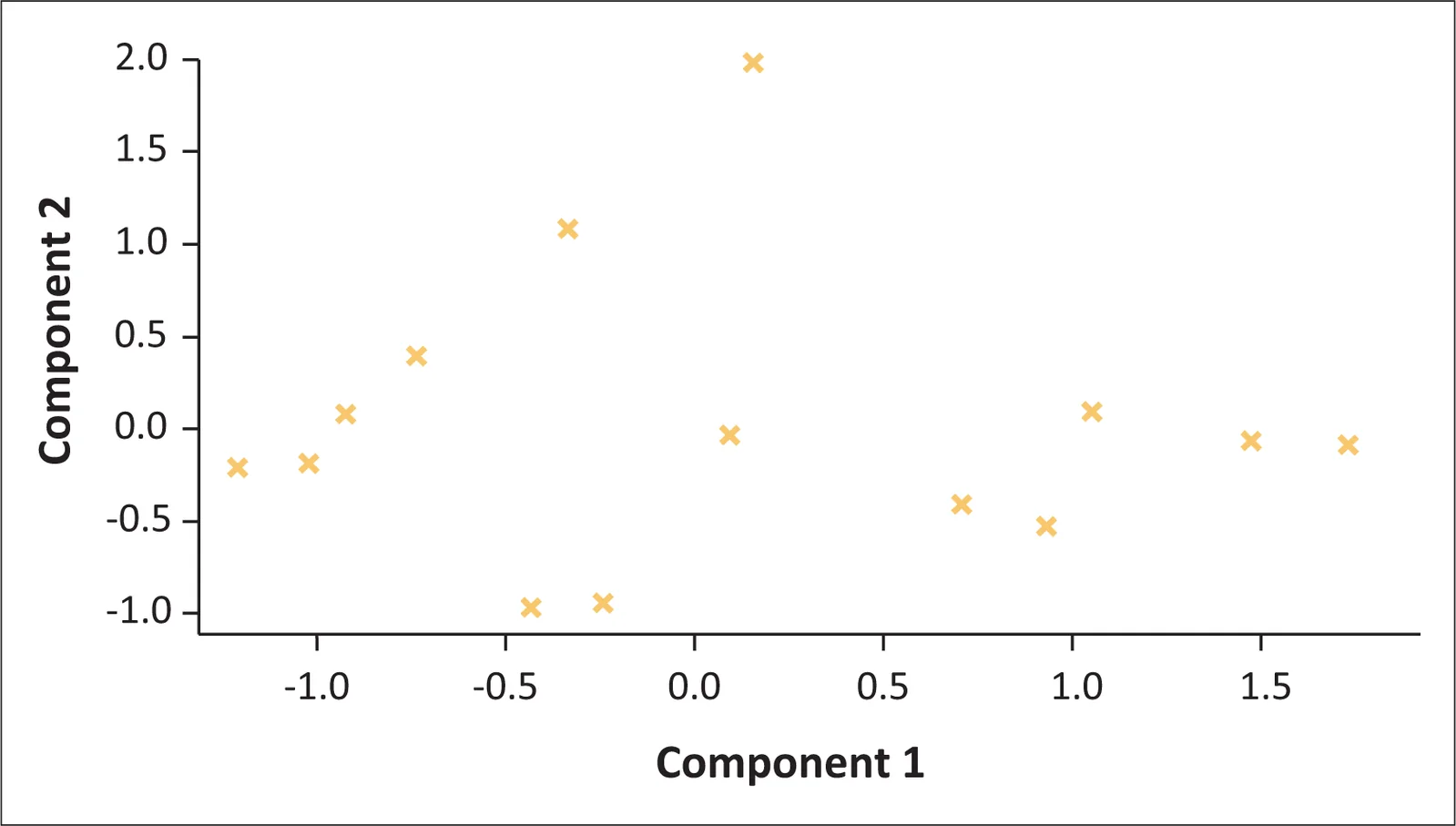
A multiple correspondence analysis proxy plot (via principal component analysis) for community risk-perception patterns.

The clustering of education and knowledge of drainage systems indicates that respondents with grade 12 or higher education are more likely to report an understanding of stormwater drainage systems. This observation supports the earlier chi-square test showing a significant association between these variables. Race and sustenance patterns suggest that mixed-race respondents are slightly more likely to report having savings or insurance, despite high unemployment. This finding implies some socio-economic variation across race. Responses indicating that the municipality is doing enough were scattered across all severity levels, suggesting a lack of consensus or inconsistent experience. Older respondents tend to report radio or no communication, while younger individuals expect more interactive formats.

## Discussion

The study assessed the patterns of flood preparedness and perception in a marginalised community of Green Point in the Sol Plaatje Municipality in the Northern Cape.

The findings of this study highlight how flood preparedness in marginalised communities such as Green Point is shaped by a combination of socio-economic and institutional factors rather than physical exposure alone. Education and employment emerged as dominant predictors of preparedness, confirming theories of social vulnerability, which posit that adaptive capacity is unevenly distributed across socio-economic strata (Fatemi et al. 2017; Rufat et al. 2019). This result supports the broader theoretical view that disaster risk is socially constructed through systemic inequality, governance capacity and public trust rather than natural hazards alone (Wisner, Gaillard & Kelman 2022).

The weak association between perceived municipal readiness and household preparedness suggests a disconnect between institutional responsibility and community agency. This relationship reinforces the literature on risk governance gaps in local disaster-management systems across Southern Africa (Byaruhanga et al. 2025; Ziervogel et al. 2016). The study advances community-based DRR theory by showing that even when residents recognise municipal limitations, they retain a willingness to participate in flood-risk reduction if empowered through accessible information and social structures such as churches and schools.

Qualitative evidence revealed that a dual narrative of frustration over municipal neglect coexists with a recognition of community behaviours such as illegal dumping. This duality demonstrates the importance of the co-production of resilience, in which both governance reform and behavioural change are necessary for sustainable preparedness. The random forest and MCA analyses add quantitative weight to these insights, illustrating that vulnerability is stratified but potentially reversible through education and communication interventions. This study contributes empirical evidence that complements existing disaster-risk frameworks by quantitatively and qualitatively linking socio-economic variables with perceptions and behavioural responses in an under-researched South African context. The results underscore the need for equity-focused DRR planning that aligns communication, infrastructure and social-support strategies with household capacities.

## Conclusion

### Recommendations

The municipality should target its disaster risk-reduction campaigns by education level, focusing on low-education areas with simplified messaging on drainage, flood preparedness and waste management. Regardless of demographics, residents feel uninformed; therefore, the municipality should use more community meetings, flyers and local radio to spread flood-risk updates.

The study would like to recommend introducing targeted micro-insurance and savings schemes for low-income households in flood-prone areas and a focused education effort for disaster risk-reduction directed at unemployed and low-education groups using appropriate media. The youth (18–35 years of age) were less prepared but showed awareness; therefore, these young people should be empowered through volunteering programmes and public works.

The municipality should upgrade drainage systems, conduct regular maintenance and implement fines for illegal dumping. Cleaning-up campaigns, environmental education drives and waste-sorting awareness should be launched. The risk-reduction plans should be co-designed through ward-level workshops, including churches, schools and civic groups. Flood preparedness roles for social structures within the municipal disaster-risk framework should be institutionalised. The municipality needs to align risk-communication methods to age, for example, short messaging systems and meetings for youth and radio for older adults. For race and resilience planning, the municipality should include race-informed socio-economic profiling in ward-level flood planning.

## References

[CIT0001] Attride-Stirling, J., 2001, ‘Thematic networks: An analytic tool for qualitative research’, *Qualitative Research* 1(3), 385–405. 10.1177/146879410100100307

[CIT0002] Braun, V. & Clarke, V., 2006, ‘Using thematic analysis in psychology’, *Qualitative Research in Psychology* 3(2), 77–101. 10.1191/1478088706qp063oa

[CIT0003] Broekman, C., Coetzee, C. & Cilliers, D., 2025, ‘Mapping pluralistic future flood risk scenarios for informal settlements in the eThekwini metropolitan municipality, South Africa’, *Natural Hazards* 121(18), 20949–20983. 10.1007/s11069-025-07581-w

[CIT0004] Byaruhanga, N., Kibirige, D. & Mkhonta, G., 2025, ‘Understanding South Africa’s flood vulnerabilities and resilience pathways: A comprehensive overview’, *Water* 17(17), 2608. 10.3390/w17172608

[CIT0005] Chiappini, H., Nieri, L. & Piserà, S., 2025, *Climate risk and bank lending in South Africa*, South African Reserve Bank Working Paper Series, pp. 1–34, Konstantin Makrelov, Pretoria, South Africa.

[CIT0006] Dodman, D., Hayward, B., Pelling, M., Broto, V.C., Chow, W., Chu, E. et al., 2022, ‘Cities, settlements and key infrastructure’, in G.-C. Delgado-Ramos & P. Romero-Lankao (eds.), *Climate change 2022: Impacts, adaptation, and vulnerability*, Contribution of Working Group II to the Sixth Assessment Report of the Intergovernmental Panel on Climate Change, p. 907, Cambridge University Press, New Zealand.

[CIT0007] Fatemi, F., Ardalan, A., Aguirre, B., Mansouri, N. & Mohammadfam, I., 2017, ‘Social vulnerability indicators in disasters: Findings from a systematic review’, *International Journal of Disaster Risk Reduction* 22, 219–227. 10.1016/j.ijdrr.2016.09.006

[CIT0008] Hair, J.F., Black, W.C., Babin, B.J. & Anderson, R.E., 2019, *Multivariate data analysis*, 8th edn., Cengage Learning, Boston, MA.

[CIT0009] Mshelia, Z.H. & Belle, J.A., 2024, ‘A systematic flood risk assessment of Bloemfontein watershed, South Africa’, *Geomatics, Natural Hazards and Risk* 15(1), 2423739. 10.1080/19475705.2024.2423739

[CIT0010] Nyam, Y.S., Modiba, N.T.S., Ojo, T.O., Ogundeji, A.A., Okolie, C.C. & Selelo, O.T., 2024, ‘Analysis of the perceptions of flood and effect of adoption of adaptation strategies on income of informal settlements of Mamelodi in South Africa’, *Climate Services* 34, 100468. 10.1016/j.cliser.2024.100468

[CIT0011] Raphela, T.D. & Matsididi, M., 2025, ‘The causes and impacts of flood risks in South Africa’, *Frontiers in Water* 6, 1524533. 10.3389/frwa.2024.1524533

[CIT0012] Rufat, S., Tate, E., Emrich, C.T. & Antolini, F., 2019, ‘How valid are social vulnerability models?’, *Annals of the American Association of Geographers* 109(4), 1131–1153. 10.1080/24694452.2018.1535887 PMC11611389 39624068

[CIT0013] Wang, Y., Zhang, J. & Li, Z., 2017, ‘Urban flood risk assessment based on a GIS–AHP multi-criteria analysis method: A case study of Nanjing, China’, *Natural Hazards* 89(2), 745–763.

[CIT0014] Wen, Y. & Shaw, R., 2025, ‘Social resilience to urban floods: Reflections from existing knowledge and practice’, *Urban Governance* 5(3), 303–313. 10.1016/j.ugj.2025.06.001

[CIT0015] Wisner, B., Gaillard, J.-C. & Kelman, I., 2022, *Handbook of hazards and disaster risk reduction and management*, 2nd edn., Routledge, London.

[CIT0016] Ziervogel, G., Waddell, J., Smit, W. & Taylor, A., 2016, ‘Flooding in Cape Town’s informal settlements: Barriers to collaborative urban risk governance’, *South African Geographical Journal* 98(1), 1–20. 10.1080/03736245.2014.924867

